# Pharmacokinetics, Tissue Distribution and Excretion of Demethyleneberberine, a Metabolite of Berberine, in Rats and Mice

**DOI:** 10.3390/molecules28237725

**Published:** 2023-11-23

**Authors:** Jingqi Li, Qi Zhang, Yutong Chen, Chengyu Lu, Yongbin Tong

**Affiliations:** College of Pharmacy, Guangdong Medical University, Dongguan 523808, China; jingqi_liphar@126.com (J.L.); zhangqilucky520@foxmail.com (Q.Z.); chyttttt@163.com (Y.C.)

**Keywords:** demethyleneberberine, pharmacokinetics, tissue distribution, excretion, rats and mice

## Abstract

Demethyleneberberine is an active component extracted from the Chinese herbal drug Cortex Phellodendri. It is also a metabolite of berberine in animals and humans. However, the pharmacokinetics, tissue distribution and excretion of demethyleneberberine have not been reported. The present study aimed to investigate the pharmacokinetic parameters of demethyleneberberine by applying high-performance liquid chromatography-tandem mass spectrometry (LC-MS/MS). After intragastric administration of demethyleneberberine in rats and mice, the pharmacokinetics, tissue distribution and excretion of demethyleneberberine were comparatively studied for the first time. The plasma concentration of demethyleneberberine reached its peak within 5 min after intragastric administration in both rats and mice. Furthermore, its bioavailability was comparable, ranging from 4.47% to 5.94%, higher than that of berberine. The total excretion of demethyleneberberine in the urine, feces and bile was 7.28~9.77%. These findings provide valuable insights into the pharmacological and clinical research on demethyleneberberine.

## 1. Introduction

Demethyleneberberine is one of the main metabolites of berberine in animals and humans [1]. Berberine and demethyleneberberine are the main active components of Cortex Phellodendri, which is a traditional Chinese medicinal material derived from the bark of the Rutaceae plant, Winged Bark. This plant is commonly used for medicine and food. It can effectively promote the secretion of bile and pancreatic juice, facilitating the rapid excretion of bilirubin from the body. Thus, it can be used to treat and protect against liver diseases [2]. Moreover, Phellodendron extract also exhibits antibacterial [3,4] and anti-inflammatory activities [5,6,7] and demonstrates various pharmacological effects, such as reducing the risk of diabetes [8] and protecting from gastric ulcers [9].

In recent years, numerous studies have shown that berberine has a variety of pharmacological activities, including antibacterial [10] and anti-inflammatory [11,12]. Additionally, research has increasingly indicated that berberine can effectively lower blood lipids to reduce the incidence of cardiovascular diseases [13,14] and prevent the development of atherosclerosis [15,16]. Interestingly, the gut microbiota plays a crucial role in mediating the cardiovascular protection provided by berberine [17,18,19]. Importantly, berberine has demonstrated promising potential effects in various areas, such as anti-cancer [20,21,22], anti-diabetic [23,24,25], anti-rheumatoid [26], anti-obesity [27,28] and pro-bone regeneration [29,30]. It also exhibits certain protective effects on the liver [31,32], lung function [33,34] and nerves [35,36]. However, studies have found that despite berberine’s significant therapeutic potential, its plasma bioavailability is extremely low, ranging from around 0.37% to 0.68% [37,38]. First-pass elimination and malabsorption may be some of the factors that contribute to the low bioavailability of ingested berberine [39]. However, demethyleneberberine, the primary metabolite of berberine in vivo, has frequently been found at high plasma concentrations, which has led to increased attention on berberine metabolites. Therefore, further research is underway to explore new ways to overcome the challenge of low bioavailability of berberine.

Demethyleneberberine, a metabolite of berberine, also exhibits similar anti-inflammatory activity [40,41,42]. According to research reports, in vivo and in vitro, demethyleneberberine exhibits significant antioxidant effects and exerts hepatoprotective and anti-fibrosis effects through various pathways [43,44,45]. Additionally, demethyleneberberine has significant therapeutic effects on pulmonary fibrosis in mice [46]. Moreover, demethyleneberberine can also promote cancer cell apoptosis, inhibit cell proliferation [47,48] and performs better than berberine in interacting with LOX-5/COX-2 to improve benign prostatic hyperplasia [49]. Furthermore, demethyleneberberine can activate AMP-activated protein kinase (AMPK) and up-regulate the expression of hepatic low-density lipoprotein receptor, thereby achieving lipid-lowering effects [50,51,52]. Notably, studies at the cellular level have demonstrated that demethyleneberberine has pharmacological activity on the central nervous system through several signaling pathways (such as NF-κB, MAPK and AMPK) [53,54]. Therefore, demethyleneberberine has significant research value. However, there is still a lack of comprehensive pharmacokinetic data on demethyleneberberine absorption, distribution and excretion in animals. To fully comprehend the biological role of demethyleneberberine in vivo, our study aimed to evaluate its absorption in rats and mice via single intragastric administration and tail vein injection. Meanwhile, the tissue distribution and excretion of demethyleneberberine in rats and mice were also investigated after intragastric administration.

## 2. Results and Discussion

### 2.1. Validation of Method

**Figure 1:**
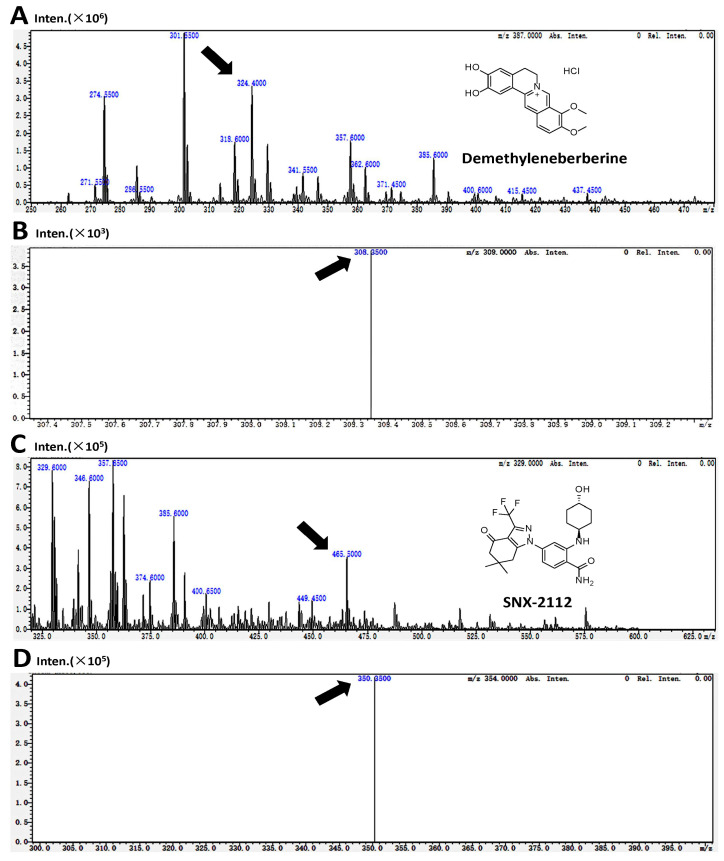
(**A**) Chemical structures of demethyleneberberine and its mass spectra of [M + H]^+^ and (**B**) its mass spectra of product ions; (**C**) chemical structures of SNX-2112 and its mass spectra of [M + H]^+^ and (**D**) its mass spectra of product ions. (Arrows: precursor and product ions of Demethyleneberberine and SNX-2112, respectively).

**Figure 2:**
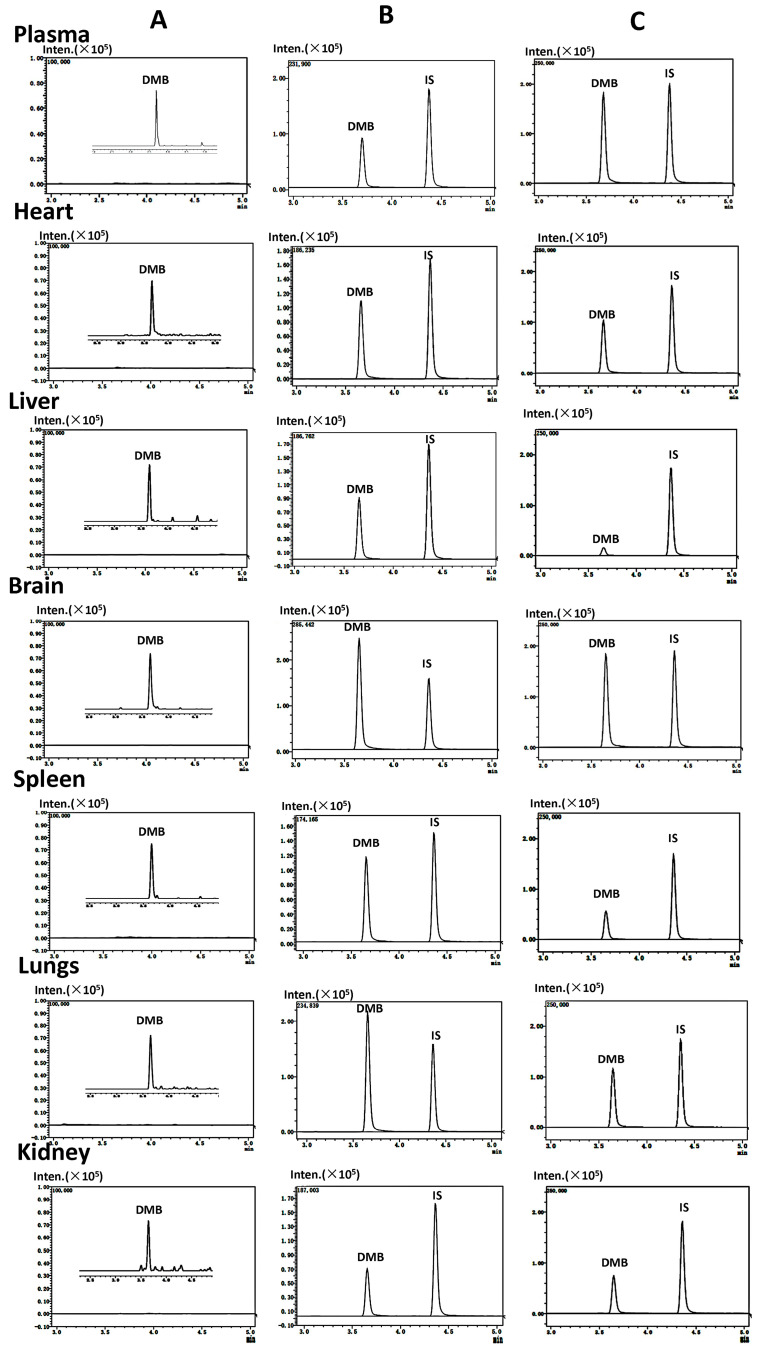

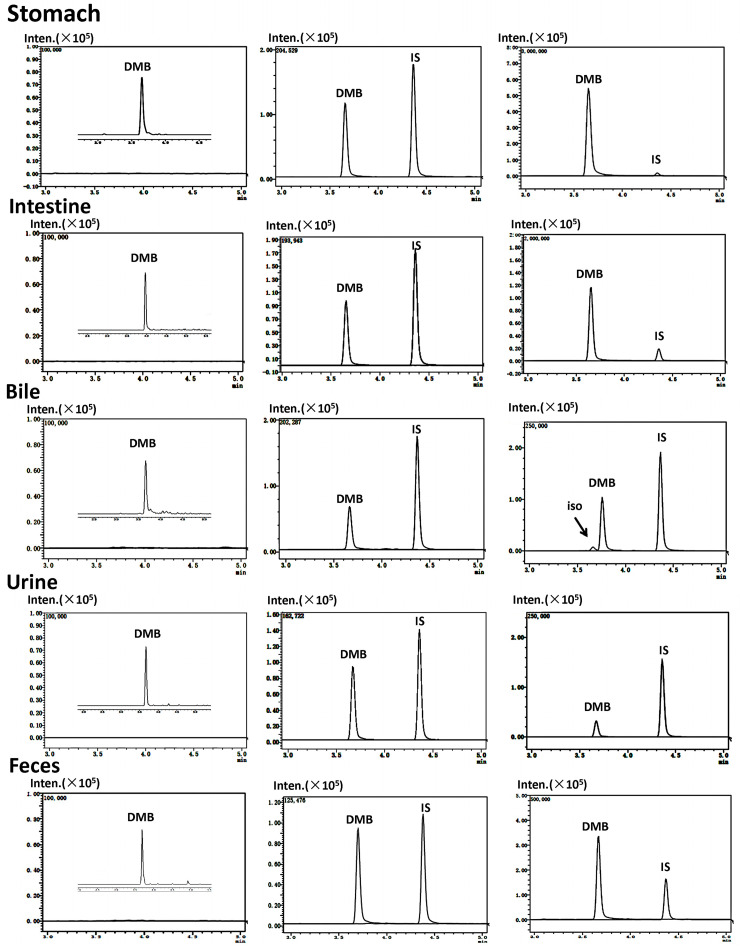
Representative extraction MRM chromatograms of demethyleneberberine (DMB) and IS in plasma, heart, liver, brain, spleen, lung, kidney, stomach, intestine, bile, urine and feces. (**A**) Blank biological samples and blank biological sample spiked with LLOQ (lower limit of quantification) of DMB in plasma (0.5 ng/mL); heart, liver, brain, spleen, lung, kidney, intestine and urine (1 ng/mL); stomach, bile and feces (3 ng/mL); (**B**) blank biological samples spiked with DMB (300 ng/mL for plasma, heart, liver, brain, spleen, lung, kidney, stomach, intestine, bile, urine and feces) and IS (40 ng/mL); (**C**) test samples obtained after intragastric administration of DMB (40 mg/kg, at 30 min for plasma, heart, liver, brain, spleen, lung and kidney; 4 h for stomach and intestine; 6 h for bile and urine; and 36 h for feces) and IS (40 ng/mL) in rats; iso–the isomer of DMB.

The detection of demethyleneberberine and SNX-2112 (internal standard, IS) was accomplished at ion pairs *m*/*z* 324.4→308.35 and *m*/*z* 465.5→350.35, respectively (Figure 1), with subsequent quantitative analysis. The research results showed that demethyleneberberine and SNX-2112 could be effectively separated, and no endogenous interfering substances were found at the retention time of demethyleneberberine or SNX-2112. The multiple reaction monitoring (MRM) mass spectrum of the blank biological sample spiked with demethyleneberberine and SNX-2112 is shown in Figure 2. The calibration curves, correlation coefficients and linear ranges of demethyleneberberine in plasma, various tissues and excreta are listed in Table 1. The standard curve formed between the peak area ratio (Y) of demethyleneberberine to SNX-2112 and the concentration (X) of demethyleneberberine was linear in all biological samples. The regression coefficients (r^2^) were all higher than 0.9911.

The lower limits of quantification (the lowest points of the calibration curves, LLOQs) of demethyleneberberine were 0.5 ng/mL in plasma, 1 ng/mL in the heart, liver, brain, spleen, lung, kidney and urine and 3 ng/mL in the stomach, intestine, bile and feces. The LLOQ samples were utilized to determine the precision of intra-day and inter-day measurements (RSD). The corresponding quality control (QC) samples at three concentration levels (10 ng/mL, 200 ng/mL, 800 ng/mL) were utilized to determine the precision of intra-day and inter-day measurements (RSD), extraction recovery, as well as the matrix effect. The method presented in this study was evaluated in plasma, tissue, bile, urine and feces. The intra-day and inter-day measurement results of demethyleneberberine are shown in Table 2. The intra-day RSD ranged from 0.49% to 10.39%, while the inter-day RSD ranged from 0.50% to 14.19%. The intra-day and inter-day accuracies of all samples were within 15%.

The extraction recoveries of all analytes in all samples were between 75.52% and 98.97%, with the RSD within 7.60%. Except for the bile, the matrix effect of all analytes in all samples ranged from 82.95% to 113.51%, with the RSD within 9.68% (Table 3). Potential inhibition of demethyleneberberine ionization by bile components or other factors should be considered. However, the matrix effect of each concentration of bile was similar, with the RSD within 1.28%, indicating that the determination of demethyleneberberine concentration in bile is not impacted and can be accurately measured. These results indicated that the method is suitable for the detection of demethyleneberberine in plasma, tissue, urine, feces and bile.

The stability study was conducted for the analysis of the extracted samples under different conditions, including room temperature, three freeze–thaw cycles, long-term (30 days) storage at −80 °C, 12 h in the auto-sampler (30 °C), and stability for 1 or 2 h on ice before extraction (Table 4). All sample concentrations, except for the extracted samples of the intestine and feces at room temperature, were less than ±15% of their nominal values. This may be due to the presence of some metabolic enzymes or intestinal microorganisms in the intestine, urine and feces, which can easily degrade demethyleneberberine at room temperature. Therefore, the determination of demethyleneberberine in biological samples requires sample pretreatment on ice and rapid processing (2 h deviation less than ± 15%), indicating that the samples are stable and can meet the requirements of the analysis.

### 2.2. Pharmacokinetic Parameters and Bioavailability

**Figure 3:**
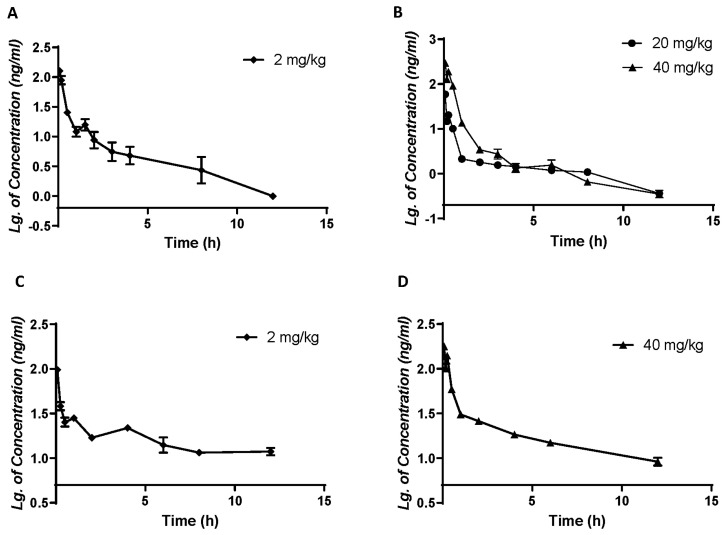
Plasma concentration–time course of demethyleneberberine (a semi–log graph) in rats after a single (**A**) i.v. dose of 2.0 mg/kg or (**B**) i.g. dose of 20 or 40 mg/kg; in mice after a single (**C**) i.v. dose of 2.0 mg/kg or (**D**) i.g. dose of 40 mg/kg. Data are presented as mean ± SD (*n* = 5 or 15).

In order to determine the oral bioavailability of demethyleneberberine, three different doses (i.v. 2 mg/kg and i.g. 20 and 40 mg/kg) were administered to rats, while C57BL/6J mice were given two different doses (i.v. 2 mg/kg and i.g. 40 mg/kg). The plasma concentration–time curves of demethyleneberberine administration were recorded and analyzed (Figure 3).

The corresponding pharmacokinetic parameters for rats and mice, fitted using non-compartmental analysis, are listed in Table 5 and Table 6, respectively. After administering 20 and 40 mg/kg of demethyleneberberine to rats intragastrically, its peak concentration (C_max_) and area under the curve (AUC_0–∞_) were 60.22 ± 12.53 ng/mL and 29.83 ± 2.14 ng·h/mL for the 20 mg/kg group and 308.25 ± 103.86 ng/mL and 133.36 ± 33.20 ng·h/mL for the 40 mg/kg group, respectively. Similarly, after administering 40 mg/kg to mice, its C_max_ and AUC_0–∞_ were, respectively, 177.15 ± 11.73 ng/mL and 264.61 ± 25.01 ng·h/mL, overall, which showed nonlinear pharmacokinetic characteristics. The intragastric (i.g.) administration of 20 mg/kg of the drug was found to have a half-life (t_1/2_) of 4.26 ± 1.48 h in rats, whereas the t_1/2_ of the 40 mg/kg group was 3.14 ± 0.42 h in rats and 4.10 ± 0.81 h in mice. Additionally, the t_1/2_ of the 2 mg/kg dose group was 5.57 ± 0.83 h in rats and 6.80 ± 1.31 h in mice.

These results suggest that demethyleneberberine is rapidly absorbed from the gastrointestinal tract and rapidly cleared from plasma. It has been reported that after intragastric administration of berberine (at a dosage of 48.2 mg/kg) [37], relatively rapid berberine absorption (T_max_, 2.75 ± 2.95 h) is observed. Demethyleneberberine is absorbed significantly faster from the gastrointestinal tract (T_max_, 0.08 ± 0.00 h) than berberine. In addition, the oral bioavailability of demethyleneberberine is relatively low, with values of 2.44% at 20 mg/kg and 5.92% at 40 mg/kg in rats and 4.47% at 40 mg/kg in mice. The results showed that the bioavailability of rats and mice is comparable. Compared with berberine (0.37~0.68%) [37,38], the bioavailability of demethyleneberberine is higher. If necessary, additional chemical structural modifications and pharmacological interventions may be performed to increase intestinal absorption and improve demethyleneberberine bioavailability. The plasma concentration–time curves of demethyleneberberine in both rats and mice have double peaks, indicating that there may be a phenomenon of enterohepatic circulation. Related studies have found that berberine metabolites can be reabsorbed through hepatic circulation, which once again confirms the phenomenon of enterohepatic circulation [1].

### 2.3. Tissue Distribution Study

**Figure 4:**
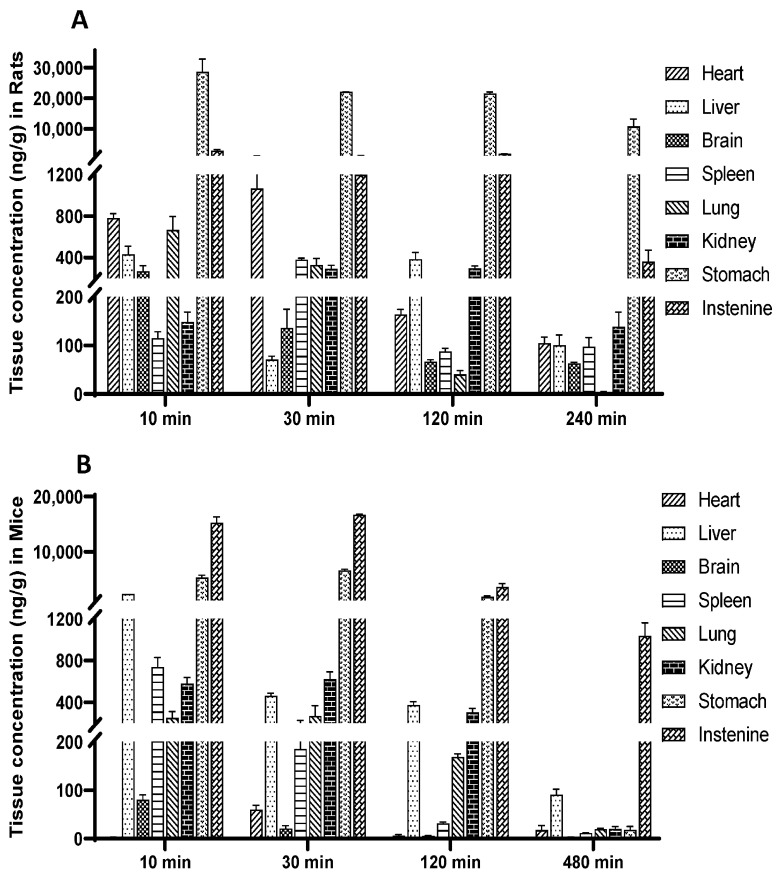
Tissue distribution of demethyleneberberine, (**A**) in rats after a single i.g. dose of 40 mg/kg; (**B**) in mice after a single i.g. dose of 40 mg/kg. Data are presented as mean ± SD (*n* = 5).

To investigate the distribution of demethyleneberberine in different tissues, we conducted a tissue distribution study in rats and mice following a single intragastric dose of 40 mg/kg. The concentration of demethyleneberberine in various tissues of rats was measured at 10 min, 30 min, 120 min and 240 min after administration. Meanwhile, the concentration of demethyleneberberine in different tissues was measured at 10 min, 30 min, 120 min and 480 min after administration in mice. The results are presented in Figure 4. The data on the plasma-to-tissue ratio can be seen in Table 7.

Demethyleneberberine was rapidly distributed to all examined tissues in rats within 10 min of administration, and its highest concentrations were detected in the stomach (28,585.86 ng/g) and intestine (2934.97 ng/g). In addition to the gastrointestinal tract, the peak concentrations of demethyleneberberine in other tissues were in the heart (1066.17 ng/g), lung (664.85 ng/g), liver (432.50 ng/g), spleen (378.39 ng/g), kidney (294.99 ng/g) and brain (267.56 ng/g). After administration in mice, the order of demethyleneberberine concentrations was as follows: intestine (15,254.14 ng/g), stomach (5347.30 ng/g), liver (2371.47 ng/g), spleen (737.43 ng/g), kidney (623.73 ng/g), lung (268.53 ng/g), brain (80.30 ng/g) and heart (60.02 ng/g).

We found that, regardless of whether in rats or mice, the C_max_ of demethyleneberberine in the digestive tract was much higher than that in other organs, indicating that the gastrointestinal tract may be the main organ of absorption. However, we cannot rule out the possibility that the higher C_max_ in the digestive tract was partly due to the presence of undigested demethyleneberberine in the lumen. The results of our experiment showed that in both rats and mice, demethyleneberberine could pass through the blood–brain barrier (BBB) and enter the brain, further confirming the pharmacological activity of demethyleneberberine in the central nervous system [54]. In rats, the concentration of demethyleneberberine in the heart peaked at 30 min and reached a lower level after 240 min, while other tissues also reached a lower level after 240 min. In mice, the liver peaked at 10 min and reached a lower level after 480 min, while other tissues reached very low levels after 480 min. Whether it was a rat or a mouse, as the administration time was prolonged, the concentration in each tissue began to decrease at 30 min after administration and had significantly decreased at 120 min. Thus, the results indicate that there is no long-term accumulation of demethyleneberberine in various tissues.

In terms of rat tissue distribution, demethyleneberberine is concentrated in the heart, lung and liver. The results showed that the demethyleneberberine concentration in the heart was high within half an hour. Previous studies have shown a cardiovascular protective effect of berberine and it is possible that demethyleneberberine also has a similar effect, but no such report has emerged [14]. Moreover, the high concentration in lung tissue may serve as a crucial factor in determining the pharmacological effects related to the lungs [46]. The changes in the concentration of demethyleneberberine in the liver over time showed a bimodal trend, supporting the bimodal phenomenon of demethyleneberberine in plasma and confirming the possible existence of enterohepatic circulation. In mice, slightly different from rats, in addition to the gastrointestinal tract, demethyleneberberine was also concentrated in the liver, spleen and kidneys, and the highest distribution in the liver (2371.47 ng/g) was more than three times that of other tissues, which may be associated with the hepatoprotective effects of demethyleneberberine, which function to treat hepatitis and prevent liver fibrosis in mice [43,44,45]. Admittedly, we cannot exclude the option that the strong metabolism of demethyleneberberine in the liver causes the first-pass effect to reduce the absorption of drugs in the plasma. Furthermore, the difference in tissue distribution between rats and mice may be caused by species differences or by inconsistent distribution of enzymes and transporters.

### 2.4. Excretion Study

**Figure 5:**
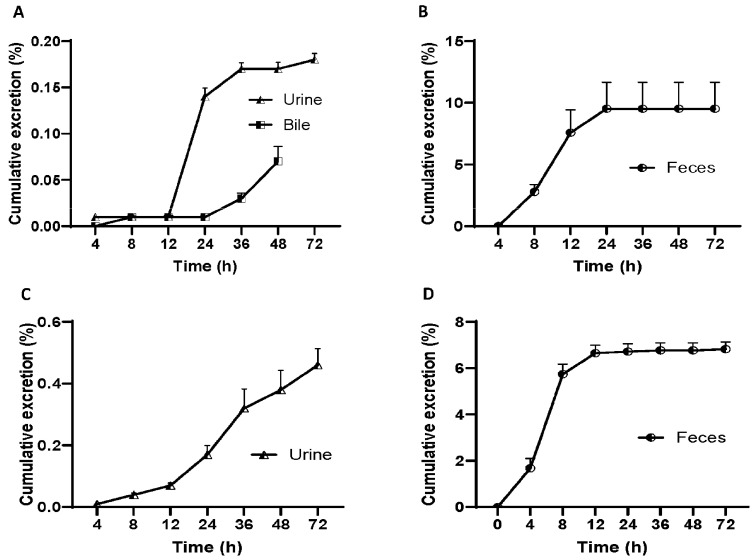
(**A**) Biliary, urinary and (**B**) fecal cumulative excretion of demethyleneberberine in rats after a single i.g. dose of 40 mg/kg; (**C**) urinary and (**D**) fecal cumulative excretion of demethyleneberberine in mice after a single i.g. dose of 40 mg/kg. Data are presented as mean ± SD (*n* = 5 or 9).

In order to study the elimination of demethyleneberberine, we conducted an experiment on rats by administering a single intragastric dose of demethyleneberberine (40 mg/kg) to measure its excretion in bile, urine and feces, and the results are shown in Figure 5.

At different time intervals, the results of the experiment demonstrated that the concentration of demethyleneberberine in urine samples peaked at 12–24 h and gradually decreased to 42 μg/mL at 36–72 h. The highest concentration of demethyleneberberine in stool samples was found at around 8–12 h. The total excretion of demethyleneberberine in urine, feces and bile was 9.77% (0.18%, 9.52% and 0.07% for urine, feces and bile, respectively). We also conducted a similar experiment on mice and measured their urine and feces. Like the results from rats, the concentration of demethyleneberberine in urine samples reached the peak at 12–24 h and gradually decreased to 163 μg/mL at 36–72 h. However, the highest concentration of demethyleneberberine in stool samples was found around 4–8 h, which was faster than observed in rats. The total excretion of demethyleneberberine in urine and feces was 7.28% (0.46% and 6.82% for urine and feces, respectively).

The results showed that the recovery rate of demethyleneberberine from rat feces was <10%, and the recovery rate from mouse feces was <8%, which was similar in both rats and mice. This indicates a lower absorption and permeability of demethyleneberberine, with the possibility that it may not be fully absorbed. Furthermore, the low blood concentration of demethyleneberberine and the low urine drug recovery rate further confirm the likelihood of malabsorption. The lower recovery rate in bile suggests that demethyleneberberine is likely to be affected by the first-pass effect, with some of the absorbed demethyleneberberine metabolized to other forms in the liver. This finding is consistent with a higher distribution in the stomach, intestine and liver. Therefore, these above results suggest that the liver may play an important role in the elimination of demethyleneberberine, but further studies are needed to determine whether drug metabolism is the main reason for the low recovery of demethyleneberberine.

## 3. Materials and Methods

### 3.1. Chemicals and Reagents

Demethyleneberberine was obtained from Dr. Tao’s laboratory [54] and SNX-2112 was purchased from Aladdin (Shanghai, China). Acetonitrile (Thermo Fisher Scientific, Waltham, MA, USA), methanol (Thermo Fisher Scientific, USA), formic acid (Macklin, Shanghai, China) and dimethyl sulfoxide (DMSO, Aladdin, China) were of chromatographically pure grade, while the rest were of analytical grade.

### 3.2. Animals

Healthy male SD rats (220–250 g) and C57BL/6 mice (18–25 g), 6 weeks old, were purchased from Guangdong Weitong Lihua Experimental Animal Technology Co., Ltd. (Foshan, China). The objective of this study was to investigate the pharmacokinetics, distribution and excretion of demethyleneberberine. All animals were maintained in a controlled environment (temperature 22 ± 3 °C, 40–60% relative humidity, 12 h light–dark cycle, 06:00–18:00). All experimental animals were housed under the above conditions for a week to adapt to the environment. Furthermore, the animals were subjected to an overnight fasting period before the experiment, while water intake remained unrestricted. The experimental protocol was approved by the National Laboratory Animal Center (Guangdong Medical University, Dongguan, China), and the care of the animals complied with the Guangdong government’s Guide for the Care and Use of Laboratory Animals. The experimental study protocol was approved by the Animal Ethics Committee of Guangdong Medical University (Dongguan, China). Ethics approval number: GDY2102041.

### 3.3. Instruments and Analytical Conditions

Samples were analyzed via LC-MS/MS-8045 (Shimadzu, Kyoto, Japan) using Labsolutions LC-MS/MS Ver.5.1 collection workstation software. The separation was performed on a Shim-pack GIST C-18 chromatographic column (2.1 × 100 mm, 3 μm). The sample was eluted with 0.1% formic acid in water (A) and acetonitrile (B). The gradient was maintained at 20% B for 1 min, then increased linearly from 20 to 90% B over 1.5 min and maintained at 90% B for 2.5 min. The temperature of the column oven was 30 °C. The flow rate was 0.3 mL/min, and the injection volume was 1 μL. The autosampler performed a needle wash for 5 s after each injection, which used acetonitrile/methanol/isopropanol/water (1:1:1:1). MS detection was performed in positive ionization mode with an electrospray ionization (ESI) source. High-purity nitrogen was used as the nebulizing and drying gas. The positive ion electrospray ionization mode was employed in the detection process using multiple reaction monitoring (MRM) technology. Other conditions were defined as follows: desolvation gas flow rate 10 L/min; ion spraying voltage 4000 V; interface temperature 300 °C.

### 3.4. Standard and Sample Preparation

#### 3.4.1. Preparation of Stock and Working Solutions

Stock solutions of demethyleneberberine (1 mg/mL) and SNX-2112 (1 mg/mL) were prepared in acetonitrile–water (50/50, *v*/*v*) and acetonitrile, respectively. To prepare the working standard solution, a series of standard solutions at 0.003, 0.01, 0.03, 0.10, 0.3, 1.00, 3.00, 10.00, 30.00 and 50.00 μg/mL was prepared by diluting the stock solution of demethyleneberberine with acetonitrile–water (50/50, *v*/*v*). The stock solution of SNX-2112 was diluted with acetonitrile to prepare a 0.04 μg/mL solution for protein precipitation. All solutions of demethyleneberberine were stored in the dark at −20 °C, and the solutions of SNX-2112 were sealed and stored at 4 °C.

#### 3.4.2. Preparation of Standard and Quality Control (QC) Samples

Appropriate amounts of working solutions were added to blank biological matrices. Three QC samples (high: 800 ng/mL; middle: 200 ng/mL; low: 10 ng/mL) were independently prepared for each sample. Standards and QC samples should be freshly prepared before use to ensure accuracy.

### 3.5. Validation of Methodology 

Methodological validation tests were performed in accordance with the currently recognized Food and Drug Administration (FDA) bioanalytical method validation guidelines [55]. For the biological matrix, we used a mixed matrix of both rats and mice. The tests included investigations of selectivity, linearity, precision, accuracy, extraction recovery, the matrix effect and stability. Each was evaluated individually to confirm the validity of the method.

The selectivity of the method was assessed by analyzing five different sources of the blank biological matrix: plasma, intestine, urine, feces and bile. The calibration curves, described as Y = aX + b, were assessed by plotting the peak-area ratios of demethyleneberberine. The lower limit of quantification (LLOQ) of demethyleneberberine was determined at the lowest point and quantifiable concentrations of the calibration curve. Precision (the relative standard deviation, RSD) and accuracy (the relative error, RE) were evaluated by analyzing QC samples at three concentration levels (low, medium and high concentrations) by studying five replicates over three consecutive days. The extraction recovery of demethyleneberberine (≥75%) was evaluated by comparing the peak areas of QC samples and control samples (extracted with acetonitrile in blank matrix and then spiked with the same concentrations). Matrix effects (deviations between 80% and 120%) were used to compare peak areas between QC samples and control samples (with water instead of matrix, while the other treatment was the same). The stability study assessed the dilution effect of the extracted samples under different conditions (deviation between 85% and 115%), including room temperature, three freeze–thaw cycles, long-term (30 days) storage at −80 °C, 12 h in the auto-sampler (30 °C), and stability for 1 or 2 h on ice before extraction.

### 3.6. Experiment

#### 3.6.1. Pharmacokinetics and Bioavailability

For the pharmacokinetic study, SD rats (five per group) were divided into three groups. The first and second groups were given 40 mg/kg and 20 mg/kg of demethyleneberberine via intragastric administration, respectively, and the third group was given 2 mg/kg of demethyleneberberine via tail vein injection. Demethyleneberberine was suspended in 5% carboxymethylcellulose sodium (CMC-Na) for intragastric administration, and it was dissolved in saline containing 10% Tween-80 and 5% DMSO for intravenous injection. After intragastric administration of demethyleneberberine, an aliquot of blood was collected from each animal at the scheduled time points: 0.083, 0.167, 0.5, 1, 1.5, 2, 3, 4, 6, 8 and 12 h. For the intravenous injection group, the scheduled time points were 0.083, 0.167, 0.25, 0.5, 1, 2, 3, 4, 6, 8 and 12 h. Blood samples were collected through the orbital venous plexus of the same animal and transferred to heparinized tubes. After centrifugation at 8000 *g* for 10 min at 4 °C, the supernatant (i.e., plasma) was collected and stored in a −80 °C refrigerator.

The mouse pharmacokinetic experiment was carried out in the same way. C57BL/6 mice (fifteen per group) were divided into two groups. The mice were administered demethyleneberberine as intragastric 40 mg/kg or intravenous 2 mg/kg. Intragastric administration was at the following scheduled time points: 0.083, 0.25, 0.5, 1, 1.5, 2, 3, 4, 6, 8 and 12 h. For the intravenous injection group, the scheduled time points were 0.083, 0.167, 0.25, 0.5, 1, 2, 4, 6, 8 and 12 h. Blood was collected through the orbital venous plexus of mice, and then we operated in the same way for rats.

We mixed 50 μL plasma sample with 250 μL acetonitrile containing 0.04 μg/mL SNX-2112 (internal standard, IS) for protein precipitation, vortexing for 2 min. After centrifugation at 12,000× *g* for 15 min at 4 °C, the collected supernatant was transferred to a new EP tube, and the solvent was evaporated using a Concentrator plus (Eppendorf, Hamburg, Germany). The dried supernatant was redissolved in 200 μL methanol/water (50/50, *v*/*v*) mixture, vortexed for 2 min and then centrifuged at 12,000× *g* for 10 min at 4 °C. The supernatant was transferred to a liquid-phase vial prior to high-performance liquid chromatography-tandem mass spectrometry (LC-MS/MS) analysis.

Pharmacokinetic parameters were calculated using DAS 2.0 software (Mathematical Pharmacology Professional Committee of China). The absolute oral bioavailability was calculated by comparing the AUC_0–t_ of demethyleneberberine after intragastric administration and intravenous injection.

#### 3.6.2. Distribution Experiment

In the distribution experiment, SD rats (five per group) were divided into four groups. Rats were intragastrically given demethyleneberberine at 40 mg/kg (suspended in 0.5% CMC-Na). After the blood was cleared via the aorta, the rats were sacrificed within 10 min, 30 min, 120 min and 240 min. The tissue samples, including the heart, spleen, lung, liver, kidney, brain, stomach and small intestine, were collected, rinsed with normal saline and blotted dry. Tissue samples were stored at −80 °C until analysis. For extraction, the organ was cut into small pieces, and it was weighed before being homogenized in 2 volumes (*v*/*w*) of normal saline. The homogenized rat tissue was ground using a JXFSTPRP-24L (Shanghai Jingxin, Shanghai, China) at 60 HZ for 120 s.

The mouse distribution experiment was carried out in the same way, with mice (five per group) divided into four groups. Mice were intragastrically given demethyleneberberine at 40 mg/kg. The mice were sacrificed within 10 min, 30 min, 120 min and 480 min. The same method was performed for the rats.

We mixed 50 μL tissue homogenate with 250 μL acetonitrile containing 0.04 μg/mL SNX-2112 for protein precipitation. Individual tissue samples were processed using similar procedures as those used for plasma samples, and then they were subjected to LC-MS/MS analysis.

#### 3.6.3. Excretion Experiments

For the excretion experiments, rats (*n* = 5) were intragastrically given demethyleneberberine at 40 mg/kg (suspended in 0.5% CMC-Na) and housed individually in metabolic cages. Urine and fecal samples were collected at the following time intervals: 0–4, 4–8, 8–12, 12–24, 24–36, 36–48 and 48–72 h. For the bile collection experiment, 5 rats were given demethyleneberberine at 40 mg/kg via intragastric administration, and the rats underwent bile duct cannulation to collect bile. Bile samples were collected at the following intervals: 0–4, 4–8, 8–12, 12–24, 24–36 and 36–48 h. Measurements were taken of the volume of urine and bile, as well as the weight of feces samples at each time point prior to extraction. Each sample was stored at −80 °C until reprocessing.

The mouse excretion experiment was conducted in the same manner as before. Nine mice were administered demethyleneberberine intragastrically at a dosage of 40 mg/kg. They were randomly divided into three groups (three per group) and housed in metabolic cages. Urine and stool samples were collected at the following intervals: 0–4, 4–8, 8–12, 12–24, 24–36, 36–48 and 48–72 h. Each sample was stored at −80 °C until reprocessing.

Fecal samples were allowed to dry before pulverization, and they were weighed before being homogenized in 5 volumes (*v*/*w*) of 50% methanol aqueous solution. The fecal samples were ground at 60 HZ for 120 s. Then, they underwent ultrasonic extraction for 10 min and centrifugation at 12,000 *g* for 15 min at 4 °C, and the supernatant was collected and transferred to a new tube. We mixed 50 μL sample solution with 250 μL acetonitrile containing 0.04 μg/mL SNX-2112 for protein precipitation. Individual tissue samples were processed using similar procedures as those used for plasma samples, and then they were subjected to LC-MS/MS analysis.

## 4. Conclusions

In conclusion, our study established and verified the use of the LC-MS/MS method for the determination of demethyleneberberine, and we investigated the pharmacokinetics, tissue distribution and excretion of demethyleneberberine in rats and mice. This study provides a reliable and cost-effective method for the determination of demethyleneberberine in a variety of biological matrices. The pharmacokinetic study investigated the concentration of demethyleneberberine at different time intervals after intragastric administration or intravenous injection. The absolute oral bioavailability of demethyleneberberine in rats ranged from 2.44% to 5.92%, and that in mice was 4.47%. Tissue distribution analysis revealed rapid and widespread distribution of demethyleneberberine in the tissues of both rats and mice. In rats and mice, a significant amount of demethyleneberberine was distributed in the gastrointestinal tissue, which showed that demethyleneberberine may be absorbed through the gastrointestinal tissue. Moreover, in addition to the gastrointestinal tract, demethyleneberberine was found to be primarily concentrated in the heart, lungs and liver of rats and in the liver of mice. In addition, less than 10% of demethyleneberberine was excreted in bile, urine or feces. Collectively, the present findings enhance our understanding of the biological functions of demethyleneberberine in vivo. These insights can be helpful not only in furthering our understanding of the potential applications of demethyleneberberine in treating certain diseases, but the research also plays a critical role in evaluating whether the in vitro effects of demethyleneberberine can be successfully translated in vivo.

## Figures and Tables

**Table 1:** Regression equations, correlation coefficients and linear ranges of demethyleneberberine in different mixed matrices of rats and mice.

Matrix	Regression Equations	Correlation Coefficient (r^2^)	Linear Range (ng/mL)
Plasma	Y = 227.71 X + 9.788	0.9984	0.5–1000
Heart	Y = 867.95 X + 15.193	0.9994	1–1000
Liver	Y = 770.17 X + 6.9004	0.9998	1–1000
Spleen	Y = 271.01 X + 16.991	0.9940	1–1000
Lung	Y = 239.7 X − 6.2526	0.9983	1–1000
Kidney	Y = 560.53 X + 13.146	0.9964	1–1000
Brain	Y = 198.63 X + 17.806	0.9948	1–1000
Stomach	Y = 418.44 X − 26.683	0.9976	3–3000
Intestine	Y = 1379.2 X + 9.7943	0.9961	1–3000
Urine	Y = 363.85 X + 12.913	0.9998	1–3000
Feces	Y = 605.06 X − 61.477	0.9930	3–5000
Bile	Y = 716.2 X − 18.949	0.9911	3–5000

**Table 2:** Intra-and inter-day precision and accuracy of demethyleneberberine in plasma, heart, liver, brain, spleen, lung, kidney, stomach, intestine, bile, urine and feces (*n* = 5).

Sample	Analyte Concentration(ng/mL)	Precision RSD (%)		Accuracy (%)	
		Intra-Day	Inter-Day	Intra-Day	Inter-Day
Plasma	0.5 (LLOQ)	4.25	8.67	104.91	114.73
	10	2.57	2.58	103.11	113.43
	200	1.34	0.99	100.90	108.32
	800	0.63	0.50	102.76	102.75
Heart	1 (LLOQ)	6.67	12.30	81.60	85.70
	10	5.50	14.74	102.58	89.73
	200	8.90	2.23	102.92	114.71
	800	0.10	5.73	93.65	89.94
Liver	1 (LLOQ)	4.67	13.97	80.02	89.75
	10	1.91	6.09	90.37	91.14
	200	3.21	0.87	106.93	104.87
	800	1.63	1.62	102.44	92.49
Brain	1 (LLOQ)	5.80	5.26	83.55	85.83
	10	13.15	10.26	91.59	87.16
	200	7.74	1.82	109.08	101.39
	800	1.86	0.08	100.83	99.22
Spleen	1 (LLOQ)	7.75	5.72	80.13	100.28
	10	2.36	8.68	106.79	115.18
	200	1.57	7.17	109.16	104.90
	800	3.55	2.69	101.39	107.88
Lungs	1 (LLOQ)	10.80	11.53	115.04	101.50
	10	1.69	2.56	87.57	91.21
	200	0.29	3.20	105.11	109.10
	800	6.99	10.62	113.73	94.75
Kidney	1 (LLOQ)	1.80	2.75	89.25	81.70
	10	4.84	3.28	110.85	85.41
	200	6.13	4.51	105.01	102.76
	800	0.44	1.92	96.11	92.05
Stomach	3 (LLOQ)	2.30	12.65	104.21	89.98
	10	8.74	9.00	115.25	90.55
	200	0.82	13.75	106.09	104.61
	800	1.34	8.31	108.20	85.88
Intestine	1 (LLOQ)	5.63	13.69	83.16	86.80
	10	3.56	2.93	111.16	102.14
	200	1.62	1.29	92.58	99.45
	800	0.49	0.96	113.87	109.00
Bile	3 (LLOQ)	2.95	14.14	115.01	114.64
	10	8.94	4.27	101.63	94.98
	200	2.54	6.25	87.42	88.67
	800	3.10	2.85	89.83	94.15
Urine	1 (LLOQ)	13.26	4.59	94.24	114.01
	10	4.89	7.98	114.91	110.41
	200	2.39	2.31	102.32	97.55
	800	0.76	0.79	109.88	109.23
Feces	3 (LLOQ)	9.87	12.23	111.99	119.44
	10	10.39	14.19	100.57	92.30
	200	3.91	0.77	113.28	90.34
	800	1.29	3.81	91.85	114.27

**Table 3:** Extract recovery and matrix effect of demethyleneberberine in plasma, heart, liver, brain, spleen, lung, kidney, stomach, intestine, bile, urine and feces (*n* = 5).

Sample	Analyte Concentration (ng/mL)	Extract Recovery (Mean ± SD, %)	Matrix Effect (Mean ± SD, %)
Plasma	10	83.17 ± 7.16	113.51 ± 0.33
	200	84.46 ± 2.06	108.25 ± 3.17
	800	82.77 ± 2.30	89.22 ± 1.02
Heart	10	101.41 ± 5.11	94.47 ± 3.01
	200	89.46 ± 11.58	103.68 ± 1.65
	800	84.14 ± 0.77	82.27 ± 2.47
Liver	10	91.87 ± 8.39	90.01 ± 9.78
	200	94.43 ± 0.41	108.25 ± 0.61
	800	102.52 ± 0.11	113.83 ± 0.11
Brain	10	101.95 ± 5.92	113.36 ± 10.55
	200	94.51 ± 0.76	98.48 ± 0.92
	800	97.54 ± 3.10	100.18 ± 0.37
Spleen	10	84.67 ± 1.12	84.13 ± 1.99
	200	91.93 ± 8.83	115.10 ± 9.42
	800	91.21 ±1.44	91.79 ± 1.46
Lung	10	98.78 ± 9.37	83.71 ± 5.63
	200	98.81 ± 1.07	88.23 ± 3.25
	800	106.93 ± 6.57	86.77 ± 4.05
Kidney	10	94.31 ± 7.53	85.78 ± 6.13
	200	101.43 ± 4.12	106.40 ± 4.32
	800	97.37 ± 0.06	97.22 ± 0.06
Stomach	10	95.23 ± 6.38	104.73 ± 4.88
	200	95.80 ± 1.00	110.74 ± 1.15
	800	100.02 ± 2.31	114.25 ± 2.68
Intestine	10	80.81 ± 4.82	90.91 ± 0.83
	200	91.90 ± 2.06	94.27 ± 4.45
	800	82.95 ± 0.61	98.07 ± 3.48
Bile	10	78.92 ± 7.15	42.86 ± 2.43
	200	75.52 ± 5.12	41.36 ± 0.87
	800	76.98 ± 5.21	39.73 ± 7.19
Urine	10	90.42 ± 7.60	85.86 ± 6.40
	200	86.15 ± 1.34	83.17 ± 1.76
	800	96.66 ± 3.30	82.95 ± 1.05
Feces	10	94.82 ± 2.46	104.27 ± 3.82
	200	98.97 ± 3.65	98.62 ± 2.83
	800	97.74 ± 5.69	100.37 ± 9.68

**Table 4:** Stability of demethyleneberberine in plasma, heart, liver, brain, spleen, lung, kidney, stomach, intestine, bile, urine and feces under different storage conditions (mean ± SD, %, *n* = 5).

Sample	Analyte Concentration (ng/mL)	Autosampler (12 h, 20 °C, Mean ± SD, %)	Room Temperature (4 h, mean ± SD, %) #	Three Freeze/ Thaw Cycles (Mean ± SD, %) #	Long-Term (30 Days, −80 °C, Mean ± SD, %) #
Plasma	10	90.18 ± 6.86	106.98 ± 2.86	105.96 ± 3.61	101.04 ± 6.64
	200	95.39 ± 7.51	112.08 ± 0.90	109.78 ± 4.25	111.33 ± 2.91
	800	96.21 ± 6.64	90.91 ± 2.29	102.05 ± 3.01	97.89 ± 1.67
Heart	10	87.65 ± 6.72	91.82 ± 1.13 ^a^	86.38 ± 1.20	103.89 ± 1.96
	200	93.75 ± 1.08	86.73 ± 19.03 ^a^	105.75 ± 6.15	110.35 ± 3.29
	800	108.92 ± 0.51	87.81 ± 1.19 ^a^	97.80 ± 0.54	98.17 ± 0.42
Liver	10	100.21 ± 9.65	102.35 ± 24.01 ^b^	90.44 ± 10.00	103.27 ± 2.04
	200	98.76 ± 1.48	98.35 ± 0.13 ^b^	101.54 ± 0.36	94.91 ± 2.41
	800	85.58 ± 3.80	99.45 ± 7.97 ^b^	92.32 ± 5.20	86.91 ± 1.67
Brain	10	86.65 ± 0.65	86.85 ± 4.01	87.34 ± 9.15	90.55 ± 9.21
	200	108.93 ± 2.64	99.83 ± 0.89	113.68 ± 0.54	110.96 ± 1.63
	800	103.42 ± 1.09	98.50 ± 3.54	103.28 ± 3.76	103.52 ± 1.07
Spleen	10	111.72 ± 7.09	92.13 ± 9.72 ^b^	106.22 ± 4.62	100.58 ± 4.46
	200	94.37 ± 1.20	98.17 ± 4.63 ^b^	101.32 ± 0.89	102.60 ± 1.34
	800	102.59 ± 3.48	96.85 ± 1.65 ^b^	98.89 ± 3.23	102.10 ± 1.26
Lungs	10	104.85 ± 4.39	89.86 ± 1.96 ^b^	99.66 ± 13.91	86.66 ± 10.88
	200	102.64 ± 3.69	87.24 ± 1.24 ^b^	96.43 ± 4.28	94.10 ± 2.16
	800	90.34 ± 0.78	86.51 ± 3.78 ^b^	91.69 ± 0.80	88.74 ± 0.84
Kidney	10	85.46 ± 1.56	92.72 ± 2.23 ^a^	91.72 ± 10.85	95.12 ± 6.87
	200	101.37 ± 0.45	94.73 ± 1.85 ^a^	102.64 ± 2.14	93.83 ± 1.46
	800	96.69 ± 0.92	86.85 ± 0.53 ^a^	97.67 ± 1.37	95.22 ± 0.74
Stomach	10	100.23 ± 0.32	86.21 ± 3.67 ^a^	85.03 ± 2.29	86.16 ± 5.14
	200	105.21 ± 1.69	86.08 ± 9.96 ^a^	99.07 ± 3.83	85.53 ± 3.05
	800	88.90 ± 0.75	85.46 ± 8.89 ^a^	90.66 ± 1.24	96.97 ± 5.77
Intestine	10	98.57 ± 2.18	101.25 ± 9.02 ^a^	104.14 ± 3.61	94.96 ± 3.47
	200	104.19 ± 1.17	98.90 ± 1.51 ^a^	92.55 ± 1.47	99.91 ± 1.46
	800	101.13 ± 1.06	89.16 ± 2.78 ^a^	95.55 ± 0.51	99.35 ± 1.28
Bile	10	88.51 ± 3.93	89.00 ± 7.70	92.34 ± 12.00	85.61 ± 5.23
	200	98.53 ± 1.23	105.37 ± 4.67	88.76 ± 1.43	104.95 ± 2.91
	800	87.08 ± 1.72	87.13 ± 3.38	89.22 ± 2.85	96.97 ± 2.09
Urine	10	86.36 ± 4.20	89.14 ± 11.04 ^a^	103.46 ± 4.15	95.21 ± 3.14
	200	85.93 ± 1.33	100.78 ± 2.01 ^a^	95.55 ± 1.38	85.15 ± 1.25
	800	95.12 ± 0.76	87.80 ± 9.98 ^a^	91.00 ± 0.13	107.43 ± 0.64
Feces	10	95.15 ± 6.64	95.79 ± 12.71 ^b^	94.65 ± 6.23	113.83 ± 4.19
	200	99.11 ± 1.28	94.84 ± 4.27 ^b^	114.04 ± 13.81	85.20 ± 4.47
	800	93.67 ± 2.55	90.58 ± 1.03 ^b^	97.56 ± 3.69	93.90 ± 0.97

# Treatment of samples before extraction. ^a^ Stability on ice for 1 h before extraction. ^b^ Stability on ice for 2 h before extraction.

**Table 5:** Non-compartmental pharmacokinetic parameters of demethyleneberberine following single i.g. (20, 40 mg/kg) and i.v. (2 mg/kg) administration in rats (mean ± SD, *n* = 5).

PK Parameters	Unit	i.v. (mg/kg)		i.g. (mg/kg)
		2	20	40
AUC_0–t_	h·ng/mL	111.13 ± 39.71	27.14 ± 2.02	131.60 ± 33.12
AUC_0–∞_	h·ng/mL	158.75 ± 95.78	29.83 ± 2.14	133.36 ± 33.20
C_max_	ng/mL	128.24 ± 15.32	60.22 ± 12.53	308.25 ± 103.86
T_max_	h	0.08 ± 0.00	0.08 ± 0.00	0.08 ± 0.00
t_1/2_	h	5.57 ± 0.83	4.26 ± 1.48	3.14 ± 0.42
MRT_0–∞_	h	5.13 ± 2.91	4.13 ± 1.35	1.13 ± 0.40
Bioavailability	%	-	2.44	5.92

**Table 6:** Non-compartmental pharmacokinetic parameters of demethyleneberberine following single i.g. (40 mg/kg) and i.v. (2 mg/kg) administration in mice (mean ± SD, *n* = 15).

PK Parameters	Unit	i.v. (mg/kg)	i.g. (mg/kg)
		2	40
AUC_0–t_	h·ng/mL	226.16 ± 28.25	202.26 ± 14.02
AUC_0–∞_	h·ng/mL	343.51 ± 34.15	264.61 ± 25.01
C_max_	ng/mL	94.50 ± 17.45	177.15 ± 11.73
T_max_	h	0.08 ± 0.00	0.08 ± 0.00
t_1/2_	h	6.80 ± 1.31	4.10 ± 0.81
MRT_0–∞_	h	10.54 ± 2.15	5.29 ± 1.30
Bioavailability	%	-	4.47

**Table 7:** The plasma-to-tissue ratio (calculated for the AUC) of each tissue in rats and mice ※.

Matrix	Rats (*t* = 240 min)	Mice (*t* = 480 min)
Heart	12.65	0.74
Liver	7.66	15.17
Brain	3.00	0.39
Spleen	5.07	2.87
Lung	4.37	5.65
Kidney	7.76	10.66
Stomach	612.73	81.00
Intestine	43.36	202.98

※ $plasma-to-tissueratio=\frac{{AUC}_{0-t}(tissue)}{{AUC}_{0-t}(plasma)}$.

## Data Availability

The data presented in this study are available in this article.

## Abbreviations

LC-MS/MS: high-performance liquid chromatography-tandem mass spectrometry; LC, high-performance liquid chromatography; MRM, multiple reaction monitoring; IS, internal standard; AUC, area under curve; RSD, relative standard deviation; LLOQ, lower limit of quantification; t_1/2_, half-life; C_max_, maximum drug concentration; T_max_, time to maximum concentration.
